# Extent and Resistance Patterns of ESKAPE Pathogens Isolated in Pus Swabs from Hospitalized Patients

**DOI:** 10.1155/2022/3511306

**Published:** 2022-10-31

**Authors:** Salim S. Masoud, Anna Kovacevich, Raidah Gangji, Helmut Nyawale, Mary Nyange, Albert Ntukula

**Affiliations:** ^1^Department of Microbiology and Immunology, Muhimbili University of Health and Allied Sciences, Dar es Salaam, Tanzania; ^2^Georgetown University, Washington, DC, USA; ^3^Department of Microbiology and Immunology, Catholic University Health and Allied Sciences, Mwanza, Tanzania; ^4^Microbiology Department, Central Pathology Laboratory, Muhimbili National Hospital, Dar es Salaam, Tanzania

## Abstract

Antimicrobial resistance has persisted as a global threat with increasing associated numbers of morbidity and mortality. ESKAPE (*Enterococcus faecium, Staphylococcus aureus, Klebsiella pneumoniae, Acinetobacter baumannii, Pseudomonas aeruginosa,* and *Enterobacter* spp.) were termed by the Infectious Diseases Society of America as a group of bacteria with rapid antibiotic resistance development. The aim of the study was to describe the extent and resistance patterns of ESKAPE pathogens isolated in pus swabs from patients admitted at Muhimbili National Hospital, Tanzania. A retrospective cross-sectional study was performed in August 2019. A total of 75 admitted patients with open wounds and surgical site infections were recruited. Files were analyzed to collect microbiology laboratory data and relevant patient data. A total of 76 clinically significant bacteria were isolated of which 52 bacteria were categorized as ESKAPE pathogens. The most common bacteria isolated were 25% (*n* = 19/76) *P. aeruginosa* and 17.1% *S. aureus.* A high level of antibiotic resistance was shown in all ESKAPE and non-ESKAPE pathogens. The Gram-negative bacteria of ESKAPE pathogens were further analyzed comparing 3^rd^ generation cephalosporin and carbapenems resistance patterns. *A. baumannii* showed the highest resistance towards 3^rd^ generation cephalosporin and carbapenems. In addition, *P. aeruginosa* showed high resistance to 3^rd^ generation cephalosporins with 89.5% resistance, with *E. coli* showing high resistance to carbapenems with 50.0% resistance. The burden of ESKAPE pathogens is high in pus swabs obtained from admitted patients at Muhimbili National Hospital. The results showed high antibiotic resistance within ESKAPE and non-ESKAPE pathogens including the “last resort” antibiotics: 3^rd^ generation cephalosporin and carbapenems.

## 1. Background

Antimicrobial resistance (AMR) is a global public threat to the treatment and cure of infectious diseases. A problem that arose from over-prescription of drugs by poor administration and ineffective therapy, and the abusive use of antimicrobials without the supervision of medical personnel [1]. The World Health Organization (WHO) categorized AMR as the fifth most urgent threat to global health, ranking above high-threat pathogens including hemorrhagic fevers, highlighting the severity of AMR in the world [2]. With the growing number of multidrug resistant (MDR) bacteria, the Infectious Diseases Society of America (IDSA) [3] termed the most notorious antibiotic-resistant bacteria in an acronym, ESKAPE: *E. faecium, S. aureus, K. pneumoniae, A. baumannii, P. aeruginosa,* and *Enterobacter species*. The ESKAPE pathogen cause infections in high-risk groups of people which include immunocompromised, hospitalized, and unwell patients resulting in life-threatening infections, prolonging hospital stays, morbidity, and mortality, and increasing financial burden [3–5].

The ESKAPE pathogens have a high potential for developing and acquiring drug resistance mechanisms: drug inactivation, modification of drug binding sites, decreased intracellular drug accumulation, and redefining paradigms in pathogenesis, transmission, and treatment [6, 7]. The listed ESKAPE pathogens have an increased occurrence of developing resistance towards 3^rd^ generation cephalosporins and carbapenems. The dissemination of resistant bacteria within communities is an emerging global threat that deserves special attention [8–10].

Multiple studies report ESKAPE pathogens contributing to a large number of bacteria isolated from various specimens of different age-group patients admitted within different departments within the hospital. Benko et al., (2020) described 72.22% (*n* = 4974/6887) prevalence of ESKAPE pathogens isolated in the emergency department. *Klebsiella* spp. has low susceptibility to 3^rd^ generation cephalosporins and beta-lactams except for carbapenems whilst *A. baumannii* demonstrated high resistance to all antibiotics including carbapenems [11]. Previous surveillance in an intensive care unit (ICU) in Mexico also describes high resistance of ESKAPE pathogens to 3^rd^ generation cephalosporin and carbapenem, particularly highlighting the alarming rates of resistant *A. baumannii.* In addition, the study reported *E. coli* as a frequently isolated bacteria with high associated antibiotic resistance [4, 12].

Data on the categorization of ESKAPE pathogens in Tanzania are finite, and with the burden of emerging antibiotic-resistant organisms isolated in patients causing high morbidity and mortality rates, it is crucial to understand the degree of ESKAPE pathogens' contribution to patient infection [13, 14]. Thus, the study aimed to describe the extent and resistance patterns of ESKAPE pathogens isolated in pus swabs from patients admitted to Muhimbili National Hospital.

## 2. Methods

### 2.1. Study Design and Duration

A retrospective cross-sectional data analysis was conducted during the 2^nd^ week of August 2019 at MNH, Dar es Salaam, Tanzania. A total of 75 admitted patients with open wounds and surgical site infections (SSI) were recruited. Patient files were reviewed to collect microbiological laboratory data and relevant patient data.

### 2.2. Study Population

All patients admitted with an open wound from different wards were subjected to bacteriological processing at the Central Pathology Laboratory (CPL) for pus swab culture. A total of 75 patient pus swabs specimen data were reviewed which included swabs from the skin, traumatic wounds, surgical wounds, burns, nose, eyes, and ear discharge.

### 2.3. Data Collection

A convenience sampling methodology was implemented, and data were retrieved from in-patient files. Available patient data: age, sex, and admitted ward, and microbiological data: organism identification and antibiotic susceptibility test, were obtained.

### 2.4. Ethical Clearance

Ethical clearance number DA. 287/298/01A/ and permission MNH/TRCU/IRB/permission/2019/117 to conduct the study were obtained from the ethical committee of the Muhimbili University of Health and Allied Sciences and Muhimbili National Hospital, accordingly. Patient identification data were deidentified before data analysis to maintain utmost patient confidentiality.

### 2.5. Laboratory Procedures

All pus swabs were collected aseptically using sterile swabs on clean viable tissue, avoiding pus or necrotic tissue after initial cleaning of the wound with normal saline to remove normal flora. Trained infection prevention and control personnel were responsible to collect the specimen. Specimens collected were transported by Stuart Transport Media within 2 hours of collection. Pus swabs were inoculated on sheep Blood agar (BA) and MacConkey agar (MCA) were incubated at 37°C in ambient air for 24 hours. Colony morphology, gram-stain, and conventional biochemical tests were applied to identify isolated bacteria. Biochemical tests for Gram-positive bacteria: catalase and coagulase tests; for Gram-negative bacteria: glucose/sucrose fermentation, hydrogen sulfide production, indole production, urease production, citrate utilization, and motility. An API20E (analytical profile index test) was used for Gram-negative bacteria which failed to be identified using conventional methodology.

Antibiotic susceptibility test for significant bacteria isolates was performed using Kirby-Bauer disc diffusion and categorized according to Clinical and Laboratory Standards Institute (2019) antibiotic breakpoints. Antibiotic categories used included: carbapenems: meropenem/imipenem, *β*-lactam antibiotics (piperacillin-tazobactam), 3^rd^ generation cephalosporins: ceftazidime/cefotaxime/ceftriaxone, aminoglycoside: amikacin/kanamycin, sulfonamide: cotrimoxazole, fluoroquinolone: ciprofloxacin, and penicillin. Methicillin-resistant *Staphylococcus aureus* (MRSA) was determined by resistance to cefoxitin.

## 3. Results

**Figure 1:**
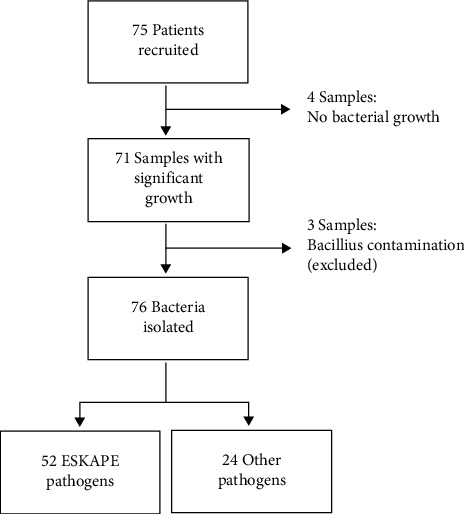
Categorization of bacteria isolates. The figure shows a schematic diagram of the overview samples eliminated and differentiation of pathogens isolated from pus swabs of admitted patients.

The study population included 75 admitted patients presenting with an open wound and SSI subjected to pus swabs. The median age of the study was 32 years (IQR; 1 day–73 years). There was an equal number of males and females accounting in the study population with 36 males and 36 females. The wards ranged from surgical ICU, surgical wards, and pediatric ICUs and wards. A total of 76 clinically significant bacteria were isolated, with 68.4% (*n* = 52/76) ESKAPE pathogens as shown in Figure 1.

**Figure 2:**
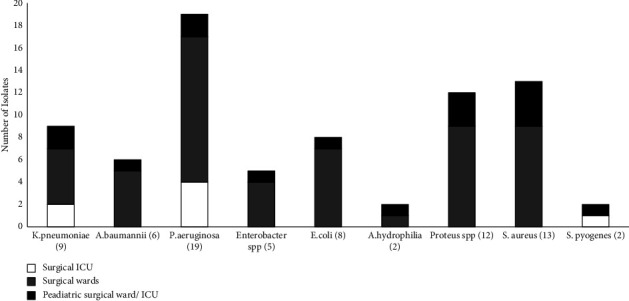
The number of bacteria isolated from different wards. *Pseudomonas aeruginosa* is the most frequent pathogen isolated commonly isolated in surgical wards. Name of bacterial isolate (number of bacterial isolates).

The most common pathogens isolated were among the ESKAPE pathogen: 25% (*n* = 19/76) *P. aeruginosa* and 17.1% (*n* = 13/76) *S. aureus*. The least common ESKAPE pathogen isolated was *Enterobacter* spp. 9.6% (*n* = 5/52) and *E.faecium* was not isolated. The most frequent ward with positive pus swab cultures was the surgical ward at 69.7% (*n* = 53/76) followed by the pediatric surgical ward/ ICU 21.1% (*n* = 16/76) as shown in Figure 2.

Figure 2 shows *P. aeruginosa* was the most frequent bacteria isolated from the surgical ward with 24.5% (*n* = 13/53) followed by *S. aureus and Proteus* spp. with 16.9% (*n* = 9/53). *P. aeruginosa* was also the most frequent bacteria isolated from the surgical ICU with 57.1% (*n* = 4/7). *S. aureus* was the most frequent bacteria isolated in the pediatric ward and ICU with 25% (*n* = 4/16).

A total of 11.7% (*n* = 8/68) patient samples had polymicrobial isolation: 87.5% (*n* = 7/8) isolated multiple Gram-negative pathogens, and 12.5% (*n* = 1/8) isolated multiple Gram-negative and Gram-positive pathogens. The most common pair in polymicrobial isolation in SSI was *P. aeruginosa* and *E.coli* 25% (*n* = 2/8).

### 3.1. Resistance Patterns

Among the antibiotic groups tested against the pathogens isolated *A. baumannii* displayed the highest resistance, with no isolate sensitive to antibiotics such as cephalosporins, aminoglycoside, and sulfonamides. *A. baumannii* isolated also displayed the highest resistance to carbapenems among the ESKAPE pathogens. *K. pneumoniae* ranked the second bacteria among the ESKAPE pathogens showing high resistance to antibiotics cephalosporin and sulfamides with 11.1% sensitivity shown in Table 1.

In the Gram-positive ESKAPE pathogens, *S. aureus* was comparatively sensitive to the antibiotic groups. However, it showed resistance to cephalosporin with 38.5% sensitivity. The highest sensitivity was shown in carbapenems and fluoroquinolones.

Among the species isolated not part of the ESKAPE pathogens, *E. coli* displayed high resistance towards all antibiotic groups listed, with the highest resistance to *β*-lactam combination, sulfonamides, and fluoroquinolones: 12.5% sensitivity. *Proteus* spp. also showed a high level of resistance to the antibiotic groups with 0.0% sensitivity for antibiotics aminoglycoside; however, sensitivity towards carbapenems and sulfonamides was displayed.

Resistance patterns of the Gram-negative bacteria among the ESKAPE pathogens list were further scrutinized comparing the resistance towards the “last resort” antibiotics used in patient care which include 3^rd^ generation cephalosporin and carbapenems. *E. coli* was also included in the analysis due to the high resistance levels shown in Table 1.

**Figure 3:**
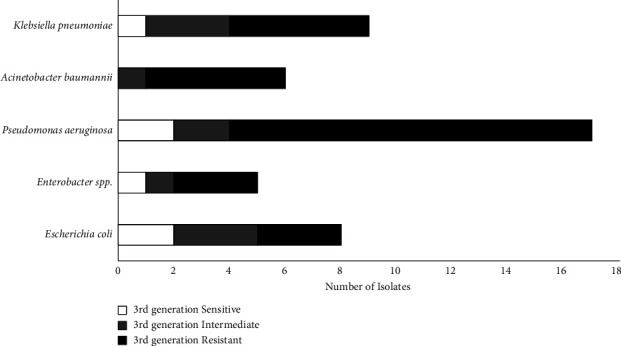
Resistance patterns of isolated gram-negative ESKAPE Pathogens. 3rd generation cephalosporins: sensitive, intermediate, and resistant were determined by CLSI 2019.

**Figure 4:**
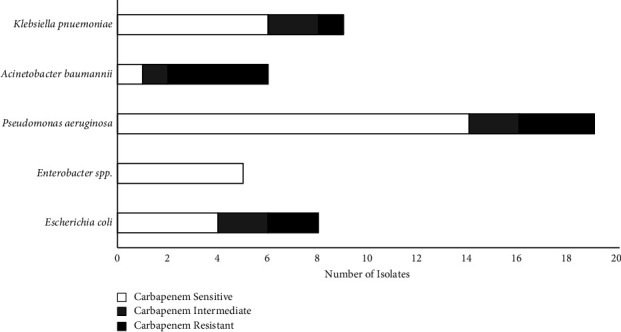
Resistance patterns of isolated gram-negative ESKAPE Pathogens. Carbapenems: sensitive, intermediate, and resistant were determined by CLSI 2019.

Comparing the 3^rd^ generation cephalosporins and carbapenems resistance patterns; 3^rd^ generation cephalosporin resistance was relatively high for all Gram-negative ESKAPE pathogens and *E. coli* showed in Figures 3 and 4. *A. baumannii* showed the highest resistance towards 3^rd^ generation cephalosporin and carbapenems compared to other pathogens included, with no isolate sensitive to 3^rd^ generation cephalosporin and 1 isolate sensitive to carbapenems. This is followed by *E. coli* showing high resistance towards 3^rd^ generation cephalosporin with 25.0% sensitivity and carbapenems with 50.0% sensitivity.

*P. aeruginosa* was the most frequent bacteria isolated, comparatively showing high resistance towards 3^rd^ generation cephalosporin but high sensitivity towards carbapenems. In addition, *Enterobacter* spp. were 100.0% carbapenem sensitive.

## 4. Discussion

Gram-negative bacteria were predominantly isolated in the pus swabs with 80.2% (*n* = 61/76). The most frequent bacteria isolated were *P. aeruginosa* 25% (*n* = 19/76), *S. aureus* 17.1% (*n* = 13/76), *Proteus* spp. 15.8% (*n* = 12/76), and *K. pneumoniae* 11.8% (*n* = 9/76). A study for SSI by Manyahi et al. analyzed drug resistant bacteria causing SSI in Tanzania, revealing the most common bacteria isolated were *P. aeruginosa* 16.3% (*n* = 24/147), *S. aureus* 12.2% (*n* = 18/147), and *K. pneumoniae* 10.8% (*n* = 16/147), aligning with this study [15]. In contrast, pathogens involved in SSI in Kenya, where the predominant bacteria are Gram-positive *S. aureus* [16]. Multiple factors can account for the variability in the predominant bacteria isolated, namely the site of the wound, immunocompromised status, comorbidities, and geographical location [17]. However, the underlining similarity between the studies is the high frequency of isolation of ESKAPE pathogens.

Bacteria commonly reported in wound infection and SSI were *P. aeruginosa, K. pneumoniae, S. aureus, E. coli, A. baumannii, Proteus* spp. [15, 17]. This study isolated uncommon bacteria such as *Aeromonas hydrophilia* and *Streptococcus pyogenes* which infrequently cause human infections. The isolation of *S. pyogenes* is infrequently isolated in human infections, mostly isolated in neonates. Surveillance of AMR in hospitals in Tanzania revealed 0.0005% (*n* = 4/7617) of *S. pyogenes* were isolated, terming the bacteria uncommon [18].

Surgical wards had the highest number of pathogens isolated with 69.7% (*n* = 53/76), with the most frequent bacteria isolated *P. aeruginosa* 24.5% (*n* = 13/53). It is recorded that the burden of wound infections is high in hospitalized patients in the ICU compared to regular wards [19–21]. However, the high number of bacteria isolated from surgical wards can be attributed to overcrowding and understaffing in hospitals, resulting in poor infection control practices [22]. This suggests the presence of nosocomial infection within the surgical ward.

The study also reported a high level of resistance among pathogens isolated from pus swabs of patients admitted to MNH. The highest resistance among all the pathogens isolated in the study was displayed by *A. baumannii*. Among the remaining ESKAPE pathogens, high resistance was displayed in *K. pneumoniae* and *P. aeruginosa*. For the non-ESKAPE pathogens, high resistance was prominent in *E. coli* isolates. A total of 17.1% (*n* = 13/76) of *S. aureus* were isolated in the study, with 7.7% (*n* = 1/13) categorized as MRSA and the remaining MSSA. There is a low reported MRSA prevalence in Tanzania which corresponds with the results of this study. In 2012, a study aimed to characterized MRSA from wound infections from a total of 26.7% (*n* = 160/600) *S. aureus,* of which 15% were MRSA [23]. In 2018, a study aimed to determine the nasal carriage of MRSA among healthcare workers reporting the overall prevalence of MRSA carriers was 15.6% (*n* = 59/379) [24]. In contrast to other East African countries, Tanzania has low levels of reported MRSA carriers isolation from clinical specimen [17, 22, 25, 26].

Resistance patterns of Gram-negative bacteria of ESKAPE pathogens towards 3^rd^ generation cephalosporins and carbapenems were further analyzed. *A. baumannii* showed the highest resistance to both 3^rd^ generation cephalosporins (0.0% sensitivity) and carbapenems (16.7% sensitivity) among all the bacteria compared. *E. coli* also showed high resistance to the antibiotic groups: 3^rd^ generation cephalosporin (25.0% sensitivity) and carbapenems (50.0% sensitivity). The findings of this study accord with other studies identifying *A. baumannii* and *E. coli* as being notorious for building carbapenem resistance [15, 17]. According to other studies, a significant proportion of *A. baumannii* infections are reported as nosocomial infections with the ability to develop antibiotic resistance [4, 20, 21].

*Enterobacter* spp. and *P. aeruginosa* were comparatively sensitive to carbapenems; however, showed resistance towards the 3^rd^ generation of cephalosporins. *P. aeruginosa* resistance to 3^rd^ generation cephalosporin is attributed to the intrinsic resistance present in the bacteria causing high production of extended-spectrum *β*-lactamases (ESBLs) [8, 27, 28]. The low carbapenem resistance in *P. aeruginosa* also concurs with other studies isolated from SSI [13, 15].

### 4.1. Study Limitation

Determination of ESKAPE pathogens into ESBLs-producing *Enterobacteriaceae* (ESBL-PE) and Carbapenemase producing *Enterobacteriaceae* (CPE) would have provided further information aiding in categorizing appropriate antibiograms for pathogens isolated from the pus swabs. In addition, the small sample size limits the study for generalization and recommendation. The MDR bacterial infection caused by ESKAPE pathogens can vary depending on the duration of hospital stays, clinical indication, previous antibiotic use, previous MDR bacterial infection, and immunocompromised status of an individual which was not collected.

## 5. Conclusion

The burden of ESKAPE pathogens is very high in pus swabs obtained from admitted patients at MNH with the most frequent bacteria isolated being *P. aeruginosa at* 25.0% (*n* = 19/76) followed by *S. aureus at* 17.1% (*n* = 13/76). Among the non-ESKAPE pathogens, 15.7% (*n* = 12/76) *Proteus* spp. and 10.5% (*n* = 8/76) *E. coli* were the most common pathogens isolated. The findings of high resistance towards the “last resort” antibiotic groups: 3^rd^ generation and carbapenems; urge the surveillance of ESKAPE pathogens, including *E. coli* and stress the importance of laboratory tests to further categorize the bacteria into ESBL-PE and CPE, providing grounds for antibiotic stewardship to guide clinicians for appropriate prescription. This would limit the acceleration of MDR bacteria among admitted patients, reducing hospital stays and encouraging fast recovery. The study findings also recommend further research into different innovative and inventive techniques in combating the crisis of AMR, which are effective and efficient [29–31].

## Figures and Tables

**Table 1:** Sensitivity patterns of bacteria isolate in percentage. Carbapenem (imipenem/meropenem), *β*-lactam combination (piperacillin-tazobactam), cephalosporin (ceftazidime/cefotaxime or ceftriaxone for gram-positive bacteria), aminoglycoside (amikacin/kanamycin), Sulfonamides (cotrimoxazole), fluoroquinolones (ciprofloxacin), and penicillin. The resistant pattern includes an intermediate breakpoint according to CLSI 2019.

Antibiotic category	K.Pneumoniae	A.Baumannii	P.Aeruginosa	*Enterobacter* spp.	E.Coli	A.Hydrophilia	*Proteus* spp.	S.Aureus	*Streptococcus* species
Carbapenem	66.7	33.3	73.7	100.0	50.0	100.0	80.0	84.6	0.0
Beta-lactam combination	33.3	16.7	68.4	60.0	12.5	50.0	33.3	NT	NT
3rdCephalosporin	11.1	0.0	10.5	20.0	25.0	50.0	33.3	38.5	50.0
Aminoglycoside	33.3	0.0	31.6	0.0	25.0	0.0	0.0	NT	NT
Sulfonamides	11.1	0.0	10.5	0.0	12.5	0.0	81.3	76.9	0.0
Fluroquinolones	33.3	16.7	68.4	40.0	12.5	50.0	50.0	92.3	50.0

^*∗∗*^NT not tested. MRSA: 1 sample, MSSA: 12 samples.

## Data Availability

All data analyzed during the study are included in this published article.
